# Characteristics of calcium deposition on expanded polytetrafluoroethylene membrane as a valve substitute in the pulmonary position

**DOI:** 10.1093/icvts/ivaf115

**Published:** 2025-05-20

**Authors:** Hayato Konishi, Akiyo Suzuki, Takahiro Katsumata, Yu Fujisawa, Tetsuya Motoyoshi, Shintaro Nemoto

**Affiliations:** Department of Thoracic and Cardiovascular Surgery, Osaka Medical and Pharmaceutical University, Takatsuki, Osaka, Japan; Department of Thoracic and Cardiovascular Surgery, Osaka Medical and Pharmaceutical University, Takatsuki, Osaka, Japan; Department of Thoracic and Cardiovascular Surgery, Osaka Medical and Pharmaceutical University, Takatsuki, Osaka, Japan; Implantable Medical Device Development Department, Teijin Limited, Hino, Tokyo, Japan; Implantable Medical Device Development Department, Teijin Limited, Hino, Tokyo, Japan; Department of Thoracic and Cardiovascular Surgery, Osaka Medical and Pharmaceutical University, Takatsuki, Osaka, Japan

**Keywords:** right ventricular outflow tract reconstruction, long-term postoperative period, valve leaflet, calcification, expanded polytetrafluoroethylene

## Abstract

An expanded polytetrafluoroethylene membrane has been widely used off-label as a substitute for a pulmonary valve leaflet. However, details regarding the calcification of the membrane in human samples have not been fully described. This report observed the precise extent and distribution of calcification in the membrane. Two samples of calcified expanded polytetrafluoroethylene membranes used as pulmonary valve substitutes were taken at replacement surgery 10 and 15 years after implantation into a valved conduit or transannular patch, respectively. In addition to general histological examination, 3D micro-computed tomography imaging and scanning electron microscopy-backscattered electron imaging were performed to reveal the precise location and extent of calcium deposition in the excised valve leaflets. Along with mineralization across the immobile membrane embedded in calcified pseudointimal tissue, calcium deposits were also detected in micro-interstices of the membrane in areas not covered by calcified fibrous tissue in both specimens. Fluorine properties and specific membrane interstices structure may cause unique calcium deposition independent of the foreign body reactions, leading to transmural calcification with thick pseudointimal embedding.

## INTRODUCTION

Despite the lack of preclinical data demonstrating efficacy and safety and of compliance with International Standard Organization-5840 for cardiac valve prosthesis, 0.1-mm expanded polytetrafluoroethylene (ePTFE) membrane (Preclude Pericardial Membrane, WL Gore & Associates, INC., Flagstaff, AZ) has been widely used off-label as a substitute for a pulmonary valve leaflet sutured into a transannular patch or a hand-made valved conduit for right ventricular outflow tract reconstruction [1, 2]. Despite its significantly better reoperation-free rates in the short and mid-term after implantation compared with xenomaterials, the ePTFE leaflets lost mobility in the long term due to inevitable material deterioration, namely calcification, along with pseudointimal embedding that were similar to other materials in the end [3, 4]. However, details regarding the calcification of the ePTFE membrane in human samples have not been fully described. In this brief communication, we shared interesting observations of the precise extent and distribution of calcification in the ePTFE membrane used as a substitute for the pulmonary valve leaflet in the long term.

## PATIENTS AND METHODS

This study was approved on 23 January 2023, by the Institutional Review Board of Osaka Medical and Pharmaceutical University (No. 2023–007-2) and was conducted in accordance with the ethical guidelines for human life science and medical research. Written informed consent was obtained from the patients’ parents.

The first specimen was obtained from an 11-year-old girl who underwent Rastelli-type operation with a φ18mm hand-made ePTFE tricuspid conduit without Valsalva sinus-like structure at 1 year of age for pulmonary atresia with ventricular septal defect. The patient has outgrown the conduit, which eventually needed to be replaced.

The second specimen was obtained from a 15-year-old boy who underwent reconstruction of the right ventricle (RV)-pulmonary artery (PA) connection 14 days after birth for truncus arteriosus. As we previously reported [5], the posterior wall of the new RV-PA route was constructed by connecting a partially resected truncal tissue including both PA branches with the superior margin of the RV incision. The anterior wall of the new RV-PA route was created with a large piece of glutaraldehyde-treated autologous pericardium after suturing a bullet-shaped piece of ePTFE membrane to the inferior two-thirds margin of the RV incision as a valve leaflet substitute. The patient underwent reoperation due to significant stenosis developed between the immobile ePTFE leaflet and the juncture of the posterior RV-PA connection. Both cases underwent successful RV-PA reconstruction using a new conduit with a bovine pericardial valve bioprosthesis sewn into a graft with Valsalva sinus geometry.

**Figure 1:**
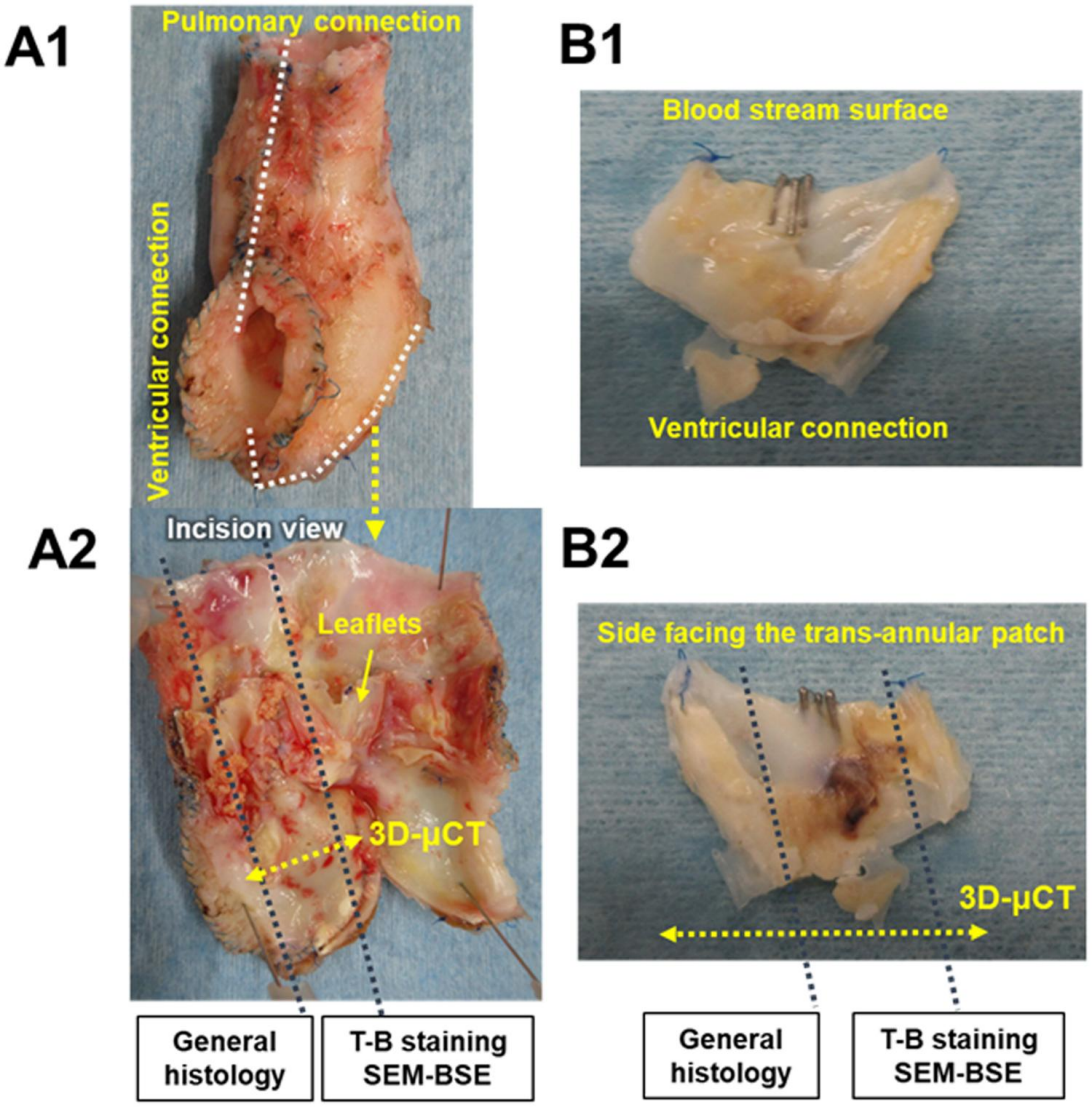
Gross appearance of the excised valved extracardiac conduit (**A**) and valve leaflet (**B**) in the pulmonary position. **A2** and **B2** show the incision line for histological analysis. μCT: micro-computed tomography; SEM-BSE: scanning electron microscopy-backscattered electron imaging; T-B: toluidine blue

The samples were fixed in 4% paraformaldehyde for later analysis. Sample sectioning and evaluation methods are shown in Fig. 1 and the Supplementary Description File, respectively. In the first case, the ePTFE tube graft of the conduit was evaluated in addition to the leaflets.

## RESULTS

The yellow thick fibrous tissue covers the leaflets extending from the attachment portion in the conduit (sample 1) or the RV incision (sample 2) (Fig. 1). All extracted ePTFE membranes were sclerotic and lost their pliability. The 3D micro-computed tomography scan revealed that the presence of calcification was consistent with the thickened fibrous tissue in the gross findings (Supplementary Video).

**Figure 2:**
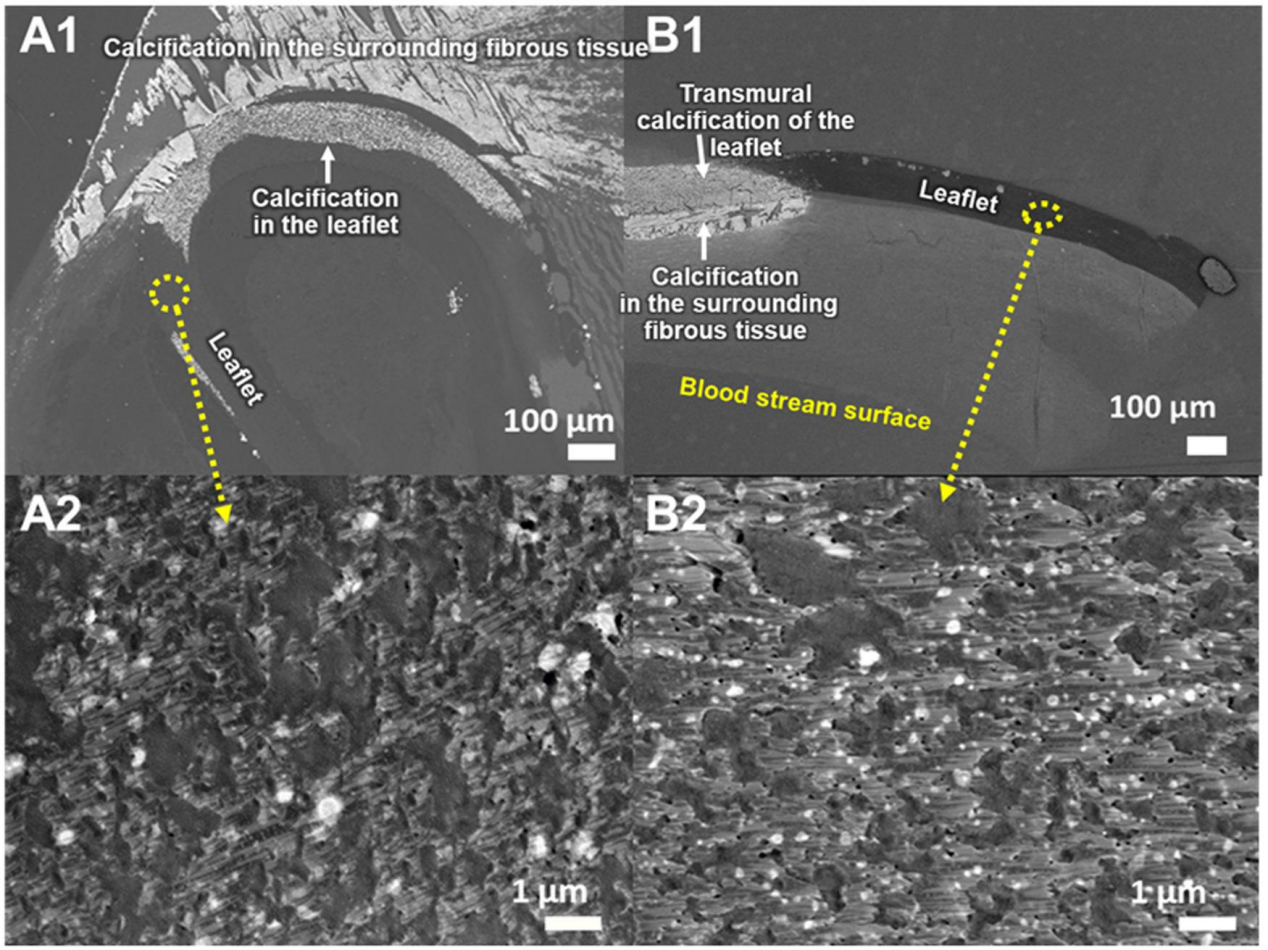
Representative photos of scanning electron microscopy-backscattered electron imaging of the excised valved extracardiac conduit (**A**) and valve leaflet (**B**) in the pulmonary position. **A1** and **B1**: low-power field; **A2** and **B2**: high-power field of the leaflet from each sample. White spots in A2 and B2 indicate calcium deposits in the micro-interstices

In the first sample from the dysfunctional conduit, routine histology showed that the leaflet was severely meandered by the proliferated collagen layers (Supplementary Fig. S1). Severe calcification and ossification in the collagen layer invades the valve leaflet. Toluidine blue staining revealed widespread calcification in the fibrous tissue on the bloodstream side and transmural calcification in the membrane. There were vacuoles infiltrating from the luminal surface into the tube graft accompanied by calcium deposition (Supplementary Fig. S3A1 and A2). Scanning electron microscopy–backscattered electron imaging (SEM-BSE) confirmed a direct connection between the two abovementioned calcifications (Fig. 2A1).

In the sample from the second case, routine histology showed mild meandering of the ePTFE leaflet and invasion of calcification from the surrounding fibrous tissue into the leaflet (Supplementary Fig. S2). The pseudointimal tissue on the bloodstream side was severely thickened and calcified next to the ventricular connection. Toluidine blue staining revealed the same findings of calcification as in the first sample. There was no cell infiltration into the leaflet membrane (Supplementary Fig. 3B1 and B2). SEM-BSE revealed the same characteristic of calcification as in the first sample (Fig. 2B1). Interestingly, at ultra-high magnification, calcium deposits inside the membrane micro-interstices were detected in areas not covered by the calcified fibrous tissue in both samples (Fig. 2A2 and B2).

## DISCUSSION

Dystrophic calcification, i.e. mineralization or crystallization, has been reported elsewhere as a well-known feature of material deterioration in ePTFE grafts used in congenital heart surgery [6]. The most likely source of minerals within the graft is intercellular calcium derived from the dissolution of cells that infiltrated and accumulated into the cavernous narrow spaces of the ePTFE graft [3–4, 6, 7]. It has also been reported that severe calcification develops along with cell death in the neo-intimal layer on the luminal side of the ePTFE graft, eventually leading to stiff and stenotic lesion [3–4, 6, 7]. The histological findings in this study confirmed the same calcification of the ePTFE tube.

On the other hand, the abovementioned cell dissolution may not occur in the ePTFE membrane because the size of the micro-interstices in the membrane does not allow cells to migrate into the membrane. One report on human samples examined by SEM suggested that ePTFE membrane calcification could be initiated by changes in the surface amorphous topography caused by proteinaceous infiltration, which triggers foreign body reactions through cell attachment [4]. On the other hand, SEM-BSE of our long-term human samples clearly revealed widespread mineral depositions inside the micro-interstices of the ePTFE membrane with a thin fibrous capsule that exhibited insignificant foreign body reaction to the membrane surface. Interestingly, in the same samples, transmural mineralization of the membrane was observed only in conjunction with significant calcification of the intimal proliferation. The internal deposition can be regarded as the innate character of the ePTFE membrane independent of the foreign body reaction, and the transmural calcification is the end-stage mineralization caused by the foreign body reaction. Considering the ionic bond character of fluoride on PTFE [8, 9], these findings suggested, at least in part, that cationic serum calcium captured by anionic fluoride on the PTFE molecules resulted in mineral formation not only on the membrane surface but also in the micro-interstices that neutralized the hydrophobicity of ePTFE, facilitating the attachment of fibrous tissue that eventually forms calcifications. The affinity of this ePTFE membrane for calcium ions was also demonstrated in *in vitro* perfusion tests [10]. Taken together, calcium deposits in the ePTFE membrane, at least, in part, may become nidus leading to extensive calcification that restricts valve motion. Although the unique extent and distribution of calcium deposition need to be confirmed by collecting and analysing more specimens of ePTFE membranes, our observational findings may also be valuable for discussion of the mechanism of calcification of ePTFE membranes used as pulmonary valve substitutes. Because there are no clinical solutions to prevent the inevitable internal calcium deposition and foreign body reaction of the ePTFE membrane at present, it would be necessary to devise the membrane attachment that prevent the substitute leaflets from embedding in the proliferated pseudointima, which restricts leaflet mobility, or to develop new calcification-resistant materials.

## CONCLUSIONS

By adding new modalities to regular histological examinations, we were able to observe unique characteristics of calcium deposition in the ePTFE membrane used for the pulmonary valve position excised in the long term.

## Supplementary Material

ivaf115_Supplementary_Data

## Data Availability

The data underlying this article are available in the manuscript.

## ABBREVIATIONS

ePTFE: Expanded polytetrafluoroethylene
PA: Pulmonary artery
RV: Right ventricle
SEM-BSE: Scanning electron microscopy–backscattered electron imaging
