# Supplementary material for: Characteristics of calcium deposition on expanded polytetrafluoroethylene membrane as a valve substitute in the pulmonary position
Source: Interdiscip Cardiovasc Thorac Surg. 2025 May 20;40(6):ivaf115. doi: 10.1093/icvts/ivaf115 (PMC12139389; doi:10.1093/icvts/ivaf115)
Supplement: ivaf115_Supplementary_Data [file ivaf115_supplementary_data.zip › Legends for Supplementary Video.docx]

**Legends for Supplemental Video**

1. First Loop: One-third of the excised valved conduit
2. Second Loop: The monocusp attached to the trans annular patch (Yellow: titanium clips placed to mimick Arantius nodule)
