# Supplementary material for: Characteristics of calcium deposition on expanded polytetrafluoroethylene membrane as a valve substitute in the pulmonary position
Source: Interdiscip Cardiovasc Thorac Surg. 2025 May 20;40(6):ivaf115. doi: 10.1093/icvts/ivaf115 (PMC12139389; doi:10.1093/icvts/ivaf115)
Supplement: ivaf115_Supplementary_Data [file ivaf115_supplementary_data.zip › Supplemental Description File.docx]

**Supplemental Description**

1. General histology

The excised tissue block was fixed with 4% paraformaldehyde. The handmade valved extra-cardiac conduit (ECC) sample was treated in an acidic decalcifying solution (#0005810, UI Kasei Corp., Hyogo, Japan) and embedded in paraffin. The transannular patch (TAP) was embedded in methyl methacrylate without decalcification. The samples were cut out, as indicated in Figures 1A2 and B2. The ECC and TAP samples were sliced using a microtome to thicknesses of 5 μm and 20-35 μm, respectively, and then stained with Hematoxylin and Eosin to examine the general status of the expanded polytetrafluoroethylene (ePTFE) membrane and graft and the biological reaction to the ePTFE.

1. 3D micro-computed tomography

Whole samples were placed and scanned under conditions of 90 kV, 100 μA for 17 s in a micro-computed tomography scanner (RmCT, Rigaku Corp., Tokyo, Japan). A 3D image of each sample was constructed using the free-software “ImageJ” (developed by Wayne Rasband, NIH. 2023).

1. Toluidine-Blue staining

Specimens were fixed with glutaraldehyde, cut into strips, washed, and re-fixed with osmium tetroxide. The strips were dehydrated, pretreated with n-butyl glycidyl ether, and re-fixed with epoxy resin. Samples in the resin block were further sliced into 1-µm sections using an ultramicrotome (EM UC7, Leica Microsystems Corp, Tokyo, Japan) and stained with 0.04% toluidine blue for 1 min. After washing the samples thoroughly, images were captured using a digital microscope (VHX-7000, Keyence Corp, Osaka, Japan). The morphological details of the cell condition and calcification were assessed by focusing on the relationship between the cell condition and the ePTFE membrane and graft.

1. Scanning electron microscopy–backscattered electron images

The toluidine-blue-stained samples were further stained with 4% uranyl acetate for 7 min, followed by 0.5% lead citrate for 5 min. The specimens were coated with 4-nm-thick osmium using a dedicated plasma coater (HPC-20, Vacuum Device Inc., Ibaraki, Japan). Backscattered Electron images were obtained using Schottky field emission scanning electron microscopy (JSM-IT800, JEOL Corp., Tokyo, Japan) at an accelerating voltage of 3.0 kV. Morphological details of mineral formation and deposition were also assessed.
