# Supplementary figures and images for: Characteristics of calcium deposition on expanded polytetrafluoroethylene membrane as a valve substitute in the pulmonary position
Source: Interdiscip Cardiovasc Thorac Surg. 2025 May 20;40(6):ivaf115. doi: 10.1093/icvts/ivaf115 (PMC12139389; doi:10.1093/icvts/ivaf115)

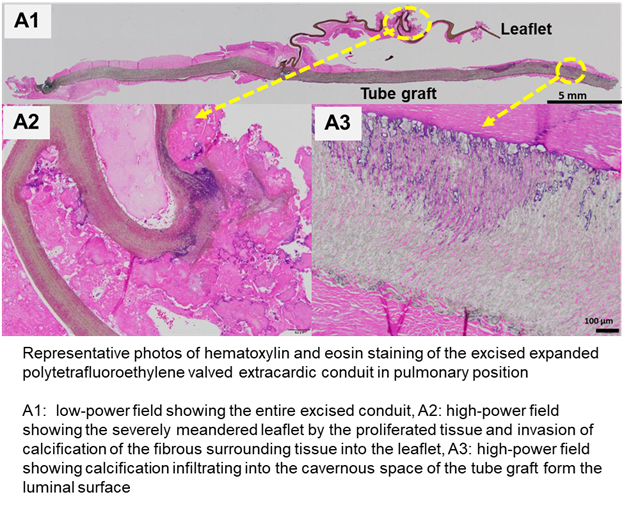

Supplement: ivaf115_Supplementary_Data [file ivaf115_supplementary_data.zip › Supplemental figure 1.tif]

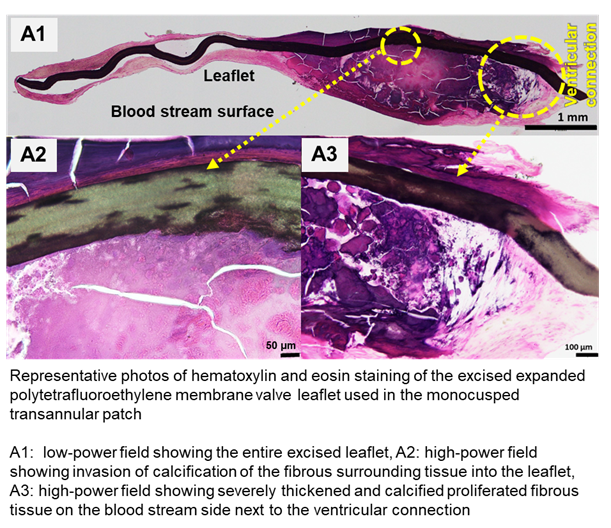

Supplement: ivaf115_Supplementary_Data [file ivaf115_supplementary_data.zip › Supplemental figure 2.tif]

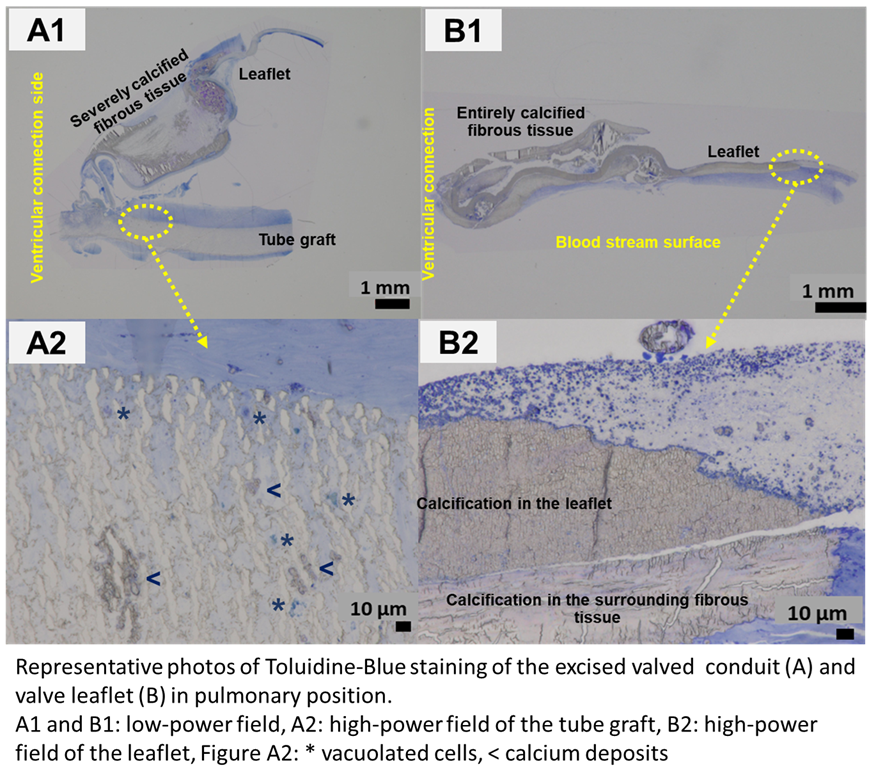

Supplement: ivaf115_Supplementary_Data [file ivaf115_supplementary_data.zip › Supplemental figure 3.tif]
